# Anatomical-based filler injection techniques for the midcheek groove and infraorbital region: Narrative review

**DOI:** 10.1016/j.jpra.2025.12.014

**Published:** 2025-12-24

**Authors:** Gi-Woong Hong, Isaac Kai Jie Wong, Jin-Hyun Kim, Kyu-Ho Yi

**Affiliations:** a Samskin Plastic Surgery Clinic, Seoul, Korea; b The Artisan Clinic, Orchard Road, Singapore; c You and I Clinic, Seoul, Korea; d Division in Anatomy and Developmental Biology, Department of Oral Biology, Human Identification Research Institute, BK21 FOUR Project, Yonsei University College of Dentistry, 50-1 Yonsei-ro, Seodaemun-gu, Seoul 03722, Korea

**Keywords:** Facial anatomy, Midface, Tear trough, Nasojugal groove

## Abstract

**Background:**

The infraorbital hollow and midcheek groove (“Indian bands”) are anatomically complex transition zones where ligamentous, vascular, and fat compartments converge. Safe and predictable correction with hyaluronic acid (HA) fillers requires precise, layer-specific anatomical understanding.

**Objectives:**

To synthesize anatomy-based injection strategies for the infraorbital–midcheek continuum, including diagnostic triage, technique selection, complication mitigation, and ethnic-specific considerations.

**Methods:**

We conducted a narrative review based on a structured search of MEDLINE, PubMed, and Ovid databases using predefined keywords related to “Dark Circle,” “Midcheek Groove,” “Indian Band,” “Dermal Fillers,” and “Facial Anatomy.” Eligible anatomical dissections, imaging-based mappings, and clinical outcome studies were qualitatively synthesized; no original patient data were collected, and no PRISMA flow diagram or quantitative meta-analysis was performed.

**Results:**

Cannula-assisted subcision to partially release fibrous retaining bands, followed by deep support (deep malar fat pad/suborbicularis oculi fat [SOOF]) and selective superficial blending, appears to improve midcheek groove correction in published series using Maili Volume and Precise. Management of overfill or surface irregularity relies on hyaluronidase and an understanding of product rheology (elastic modulus G′, cohesivity, elasticity). However, the available evidence remains heterogeneous and predominantly case-series level, with limited standardized outcomes, patient-reported measures, and long-term follow-up.

**Conclusion:**

An anatomy-based, layer-specific approach can enhance predictability and safety for infraorbital–midcheek rejuvenation, but current recommendations are largely experience-driven. Future work should prioritize controlled clinical validation, complication registries, and population-specific optimization of technique with robust, quantitative and patient-centered outcomes.

## Introduction

The aesthetic appearance of the periorbital and midfacial regions plays a crucial role in perceived facial attractiveness and age. Dark circles under the eyes and midcheek grooves, often referred to as the “Indian band,” are common concerns that can significantly impact an individual’s appearance and self-esteem.^1^ These features, while sometimes attributed to simple factors like fatigue or aging, are in fact the result of complex anatomical and physiological interactions involving skin quality, subcutaneous fat distribution, ligamentous structures, and underlying musculature.^2^, ^3^, ^4^, ^5^^,^^6^

Recent advancements in aesthetic medicine have led to a more nuanced understanding of the multifactorial etiology of these facial features. This improved comprehension has paved the way for the development of sophisticated, anatomy-based treatment approaches using dermal fillers. By targeting specific anatomical structures and addressing the root causes of these aesthetic concerns, practitioners can offer more effective and potentially longer-lasting solutions to patients seeking facial rejuvenation, while minimizing complications through respect for ligamentous and vascular landmarks.

## Methods

We conducted a narrative literature review with a structured search of MEDLINE, PubMed, and Ovid for English-language publications up to December 2024 using the terms “dark circle,” “midcheek groove,” “infraorbital hollow,” “tear trough,” “Indian band,” “dermal fillers,” “hyaluronic acid,” “facial anatomy,” and “non-surgical facial rejuvenation.““ We included anatomical dissections, imaging-based mapping studies, and clinical investigations detailing injection planes, techniques, and outcomes in the periorbital and midface regions. Studies focusing primarily on surgical approaches, animal models, or non–filler-based modalities without relevance to filler planning were excluded. Given the narrative design, we conducted a qualitative synthesis and did not perform PRISMA diagramming, risk-of-bias scoring, or quantitative meta-analysis. We acknowledge that restricting the search to English-language literature introduces language bias and that the absence of pooled effect estimates limits the strength of any comparative conclusions.

### Dark circle

#### Causes of dark circles

Dark circles under the eyes are often perceived as indicators of fatigue or sadness, contributing to a negative overall impression. These darkened areas can result from various factors beyond simple skin pigmentation. In individuals with naturally thin or pale lower eyelid skin, underlying structures such as the orbicularis oculi muscle and periorbital venous vessels become more visible, leading to the appearance of dark circles. This visibility is exacerbated by factors like fatigue and poor blood circulation, where dehydration further thins the skin and accentuates the prominence of underlying muscles and veins.^7^

Furthermore, skin laxity, including sagging of the lower eyelids, combined with morphological changes in the tear trough, orbital structures, and malar bone, as well as atrophy of the malar fat pad, can result in under-eye hollowing. These anatomical alterations create contour irregularities that emphasize the protrusion of the infraorbital fat pads, leading to shadowing and a darker appearance due to light refraction effects. Additional contributing factors include medical conditions such as sinus inflammation associated with otolaryngological issues or digestive disorders, which can cause or intensify the manifestation of dark circles under the eyes (Table 1).^3^^,^^6^

**Table 1:** Causes of infraorbital dark circles.

1. Bulging of the intraorbital septal fat with groove
2. Bulging of nasolabial & medial subcutaneous fat pads with groove or hollowness
3. Orbicularis oculi muscle (OOM) shining through thin & translucent orbital skin (same effect on lateral part of the nose near the medial canthus)
4. Facial vein on nasojugal groove under the medial muscular band of OOM
5. Subcutaneous vascular plexus, inferior palpebral vein on the periorbital region
6. Skin pigmentation
7. Medical problems (paranasal sinusitis, GI trouble, etc.)

Notably, the tear trough and midcheek groove often coexist anatomically, as both are influenced by the infraorbital fat pads and zygomatic ligaments. A unified approach to their diagnosis and treatment allows for more cohesive facial rejuvenation strategies.

#### Diagnosis and treatment based on causes

When evaluating dark circles under the eyes, it is essential to identify the underlying causes to ensure effective treatment. The following outlines the characteristics and treatment strategies according to the different etiologies.^5^

#### Pigmentation in the lower eyelid skin

– Characteristics: When dark circles are caused by dermal melanin deposition, the skin may present a brown or blue-gray discoloration. Upon applying pressure to the skin, there is no blanching or lightening, and the pigmented area may appear to spread.
– Treatment: For pigmentation-related dark circles, addressing the underlying cause of melanin deposition is essential. This may involve the use of topical treatments or laser therapy, though these approaches fall outside the scope of filler treatments.

#### Visibility of orbicularis oculi muscle or veins

**Figure 1:**
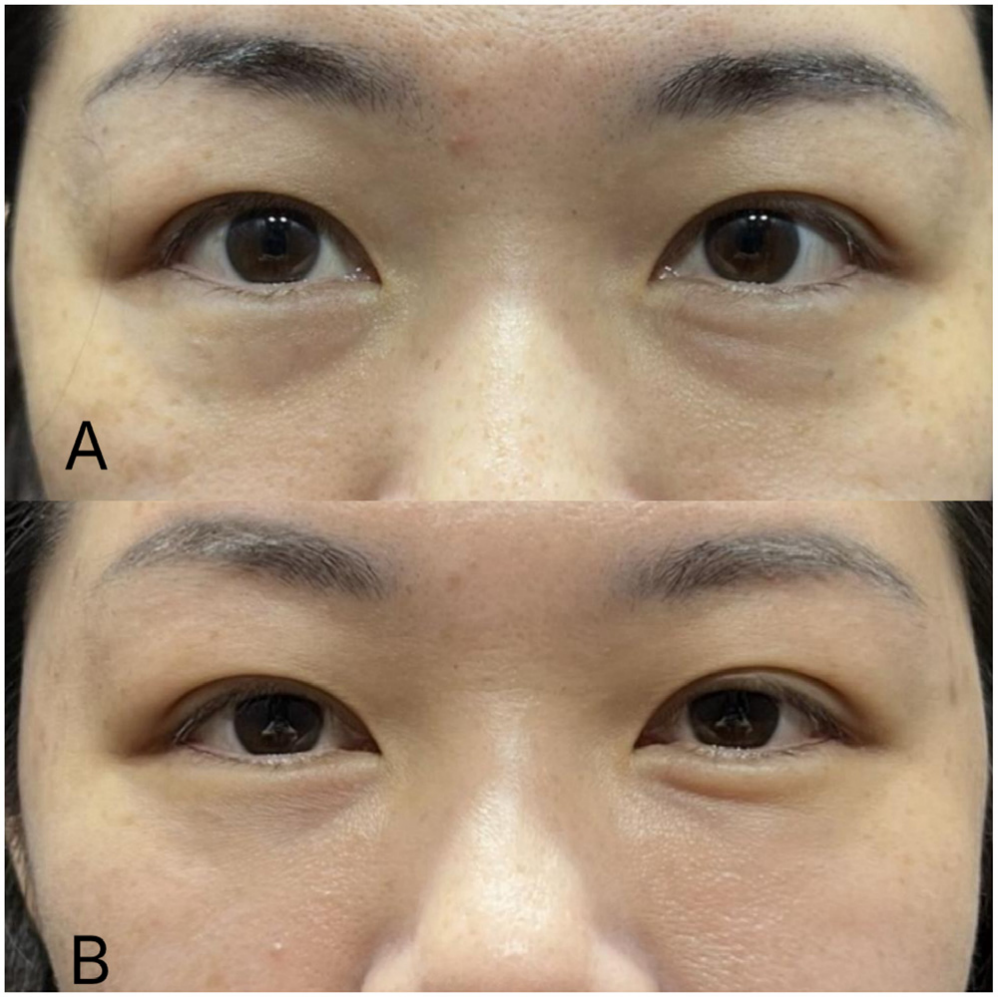
Clinical photographs demonstrating infraorbital hollowing and midcheek groove (“Indian band”) before (Panel A) and after (Panel B) filler treatment (Maili Precise, 15mg/ml, Sinclair) in a representative patient. Images published with patient consent.

– Characteristics: When the lower eyelid skin is thin and transparent, the underlying orbicularis oculi muscle or veins may become visible. The orbicularis oculi muscle often appears reddish, particularly near the nasal side of the inner canthus. The primary vein contributing to dark circles is the facial vein, which runs along the nasojugal groove beneath the medial muscular band of the orbicularis oculi muscle, often presenting as a bluish line. Additionally, prominent inferior palpebral veins or subcutaneous vascular plexuses can contribute to a bluish discoloration, which may intensify during the menstrual cycle. Pressing on the affected area does not cause blanching but rather deepens the violaceous hue.
– Treatment: When the thin lower eyelid skin permits the orbicularis oculi muscle or veins to become visible, injecting a small-particle, soft hyaluronic acid (HA) filler using the fanning technique can be an effective intervention. This method creates a barrier between the skin and the underlying structures, thereby thickening the skin and reducing the visibility of the muscle and veins. This approach is particularly effective in diminishing the appearance of redness due to muscle visibility and may also alleviate symptoms associated with deeper veins. However, it may be less effective for bluish discoloration caused by superficial venous structures. Therefore, it is crucial to accurately diagnose the primary cause of the discoloration before selecting the appropriate treatment approach (Figure 1).

As individuals age, the skin under the eyes tends to thin and, with the accompanying loss of subcutaneous fat, infraorbital ridges and hollows may develop.^4^ These changes can be further exacerbated by pseudo-herniation of the lower eyelid fat pads, which intensifies the appearance of dark circles due to shadowing effects under certain lighting conditions.^8^ Dark circles resulting from tear trough deformities, bone resorption, or malar fat pad atrophy can often be effectively treated with fillers.^2^ The treatment principles for addressing these issues align with those used for correcting infraorbital ridges and hollows.

Before initiating treatment, it is crucial to identify areas that may not respond well to filler correction. This can be achieved through specific diagnostic tests, such as the smiling test and the pulling test.

1. Smiling Test: This test evaluates the degree of lower eyelid fat pad protrusion when the patient smiles broadly. If the fat pads exhibit significant bulging during smiling and this bulging remains pronounced even when the surrounding skin is pulled tight, it suggests that additional interventions beyond fillers may be necessary to address the under-eye fat. This possibility should be communicated to the patient beforehand.
2. Pulling Test: This test assesses the degree of skin laxity by gently pulling the lower eyelid skin towards the outer corner of the eye. If the skin gathers loosely and the excess measures more than 1 cm, it indicates that non-surgical treatments may have limited effectiveness in tightening the loose skin. Demonstrating the results of this test to the patient can help them understand the extent of skin laxity and set realistic expectations for the outcomes of filler treatments.

By conducting these tests, clinicians can more effectively communicate the potential benefits and limitations of filler treatments to the patient and discuss the possibility of additional procedures that may be necessary to achieve optimal results. This approach ensures that patients have a clear understanding of the expected outcomes and are fully informed about the potential need for further interventions.

Beyond HA filler injections, treatment options for dark circles include topical bleaching agents, chemical peels, laser treatments, radiofrequency therapy, autologous fat grafting, transconjunctival fat removal, fat repositioning surgery, and lower blepharoplasty.^5^^,^^9^, ^10^, ^11^, ^12^, ^13^ A thorough understanding of the underlying causes of dark circles is essential for developing an effective treatment plan tailored to the specific etiology. Identifying the root cause enables a more targeted approach, ensuring that the selected treatment method addresses the primary contributing factors and thereby leads to more effective and durable results.

### Midcheek groove (Indian band)

#### Pre-treatment considerations for successful cheek augmentation

To achieve successful outcomes in cheek augmentation procedures, it is imperative to have a thorough understanding of the layered anatomical structures of the midface, particularly the zygomatic (cheekbone) region. The tissue layers over the zygomatic area are organized from superficial to deep as follows: skin, superficial malar fat pad, orbicularis oculi muscle, SOOF within the deep malar fat pad, facial expression muscles such as the zygomaticus muscles and levator labii superioris, preperiosteal fat, periosteum, and bone.^14^

**Figure 2:**
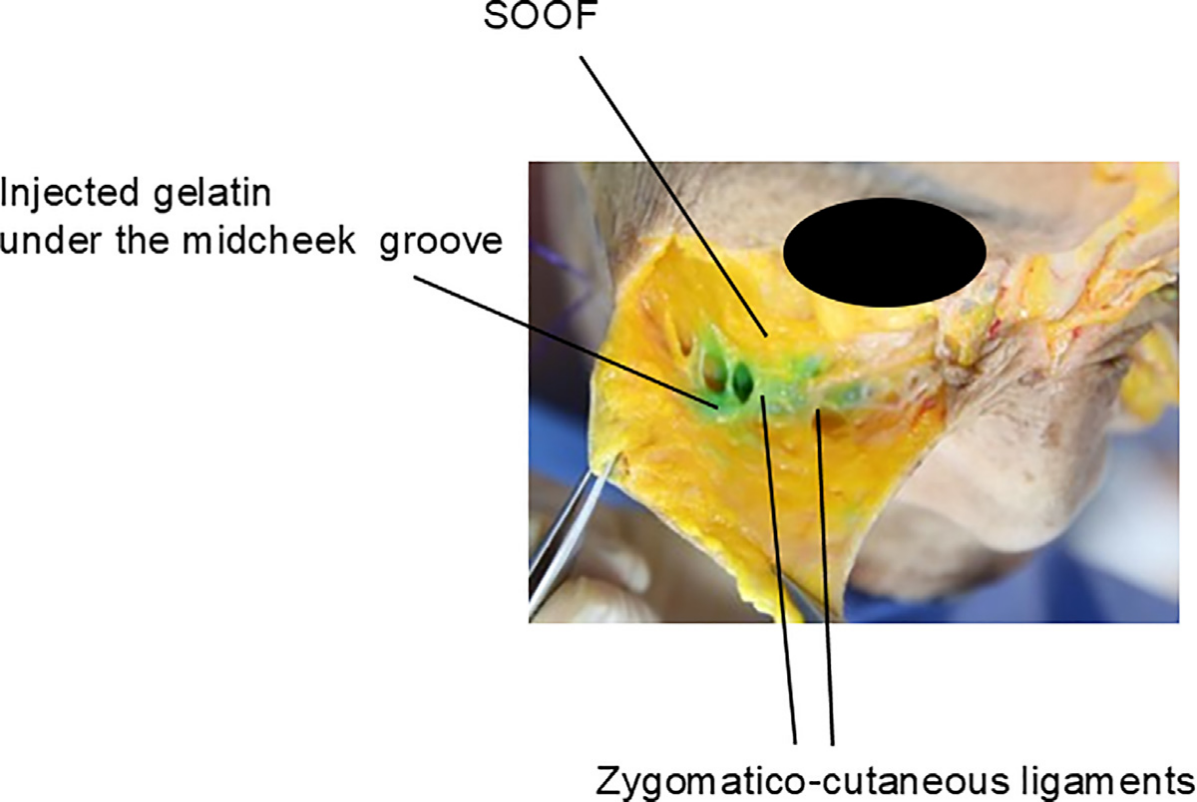
Anatomical representation of zygomatico-cutaneous ligaments in the anterior and midfacial regions. This schematic illustration delineates the precise anatomical positioning and structural composition of the zygomatico-cutaneous ligaments. These ligamentous structures are integral to facial support mechanisms and serve as primary targets in midfacial rejuvenation interventions.

**Figure 3:**
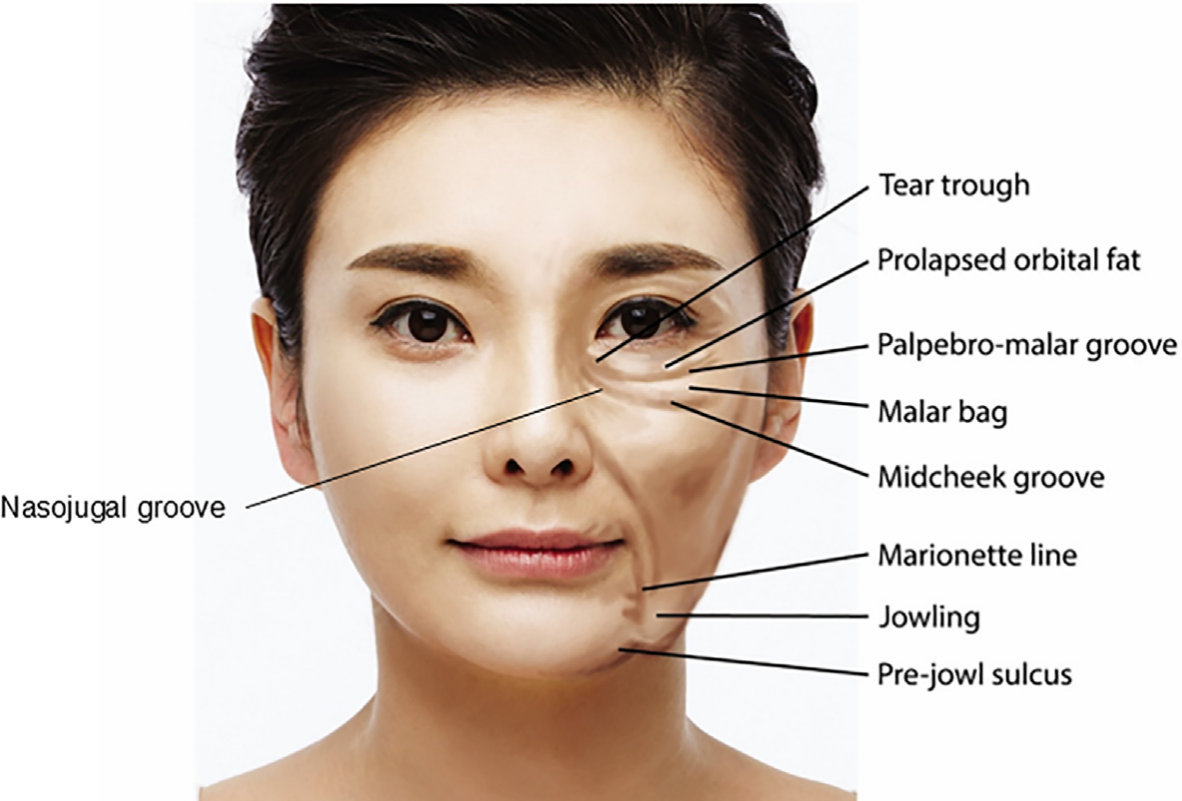
Topographical depiction of the midcheek sulcus, nasojugal groove, and tear trough deformity. This anatomical diagram offers a detailed topographical representation of key midfacial landmarks. It precisely demarcates the midcheek sulcus, nasojugal groove, and tear trough deformity, which are frequently addressed aesthetic concerns in facial rejuvenation procedures.

In this region, the zygomatico-cutaneous ligaments extend diagonally from the deeper periosteal layers to the skin, running from the upper inner portion to the lower outer part of the cheek (Figure 2). These ligaments contribute to the formation of the midcheek groove or furrow, a fibrous band that presents as a visible depression across the anterior cheek. The midcheek groove thus follows the path of the zygomatico-cutaneous ligaments as a prominent line across the cheek (Figure 3).^15^, ^16^, ^17^

**Figure 4:**
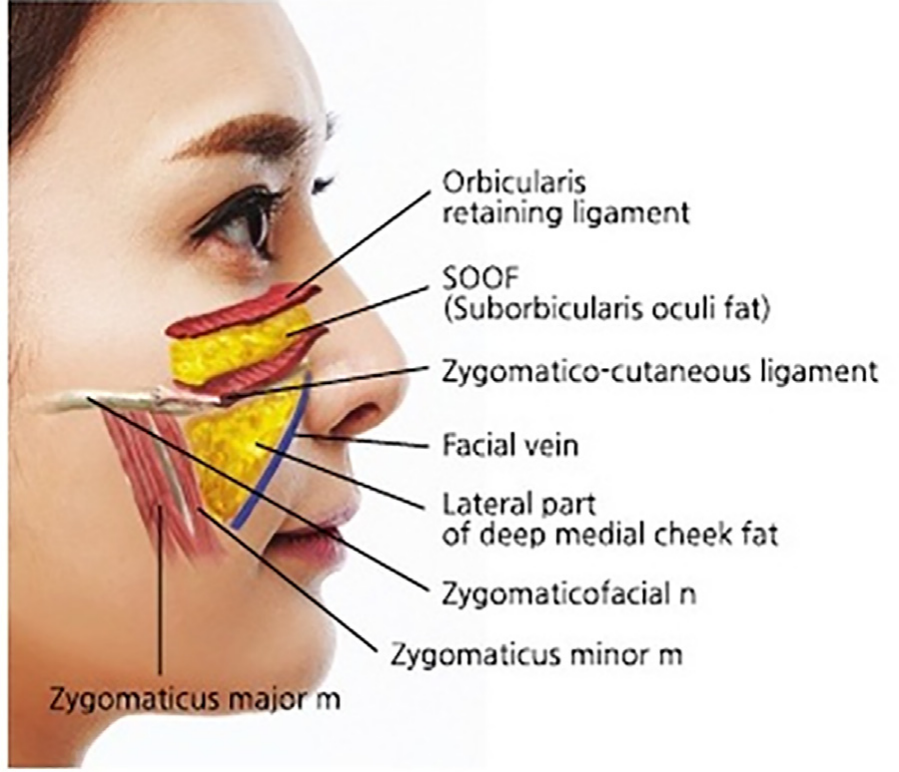
Spatial relationship between malar fat compartments and zygomatico-cutaneous ligaments. This cross-sectional illustration elucidates the intricate spatial relationship between the malar fat compartments and the zygomatico-cutaneous ligaments. It demonstrates the complex interplay of these anatomical structures in determining midfacial contour and volumetric characteristics, providing critical insights for aesthetic interventions.

The malar fat pad is divided into upper and lower segments by the zygomatico-cutaneous ligaments. When these ligaments exert significant tension, the regions above and below the band may become more prominent. As aging progresses, the deep medial cheek fat beneath the ligament, which occupies a relatively large area, tends to atrophy and become sunken. Meanwhile, the superficial fat layers, such as the nasolabial fat and medial cheek fat located above the deep fat, lose volume and support, leading to sagging. In contrast, the fat above the ligament, including the SOOF and malar fat, is stabilized by the tear trough ligament and orbicularis retaining ligament, which prevent significant sagging. This results in a noticeable contrast between the upper and lower areas (Figure 4).^18^, ^19^, ^20^, ^21^

**Figure 5:**
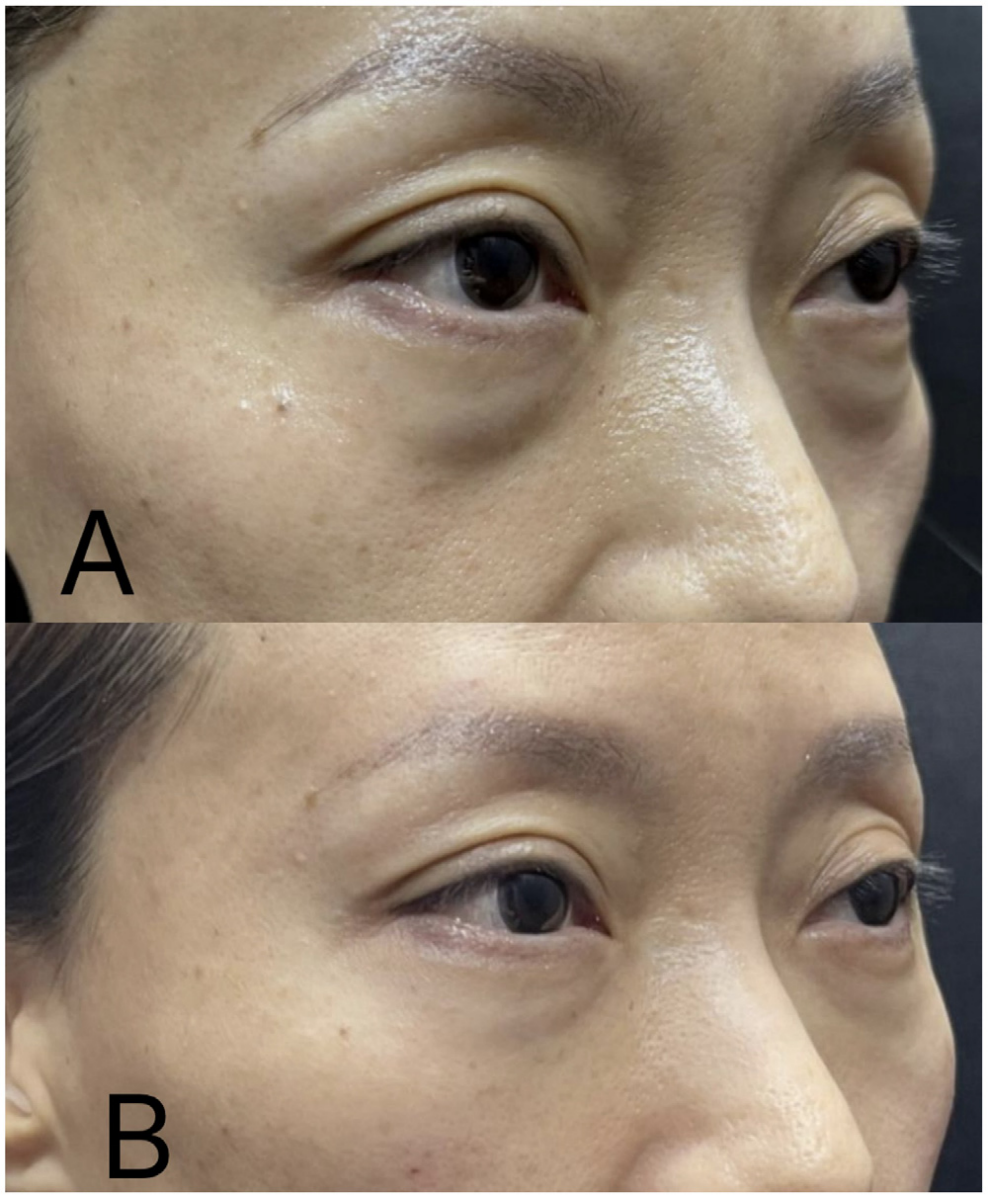
Comparative analysis of midcheek sulcus and depression pre- and post-intervention. Panel A: Pre-intervention status of the midcheek region, displaying notable sulcus depth and volumetric deficiency. Panel B: Post-intervention result (supraperiosteal treatment with Maili Volume and subcutaneous treatment with Maili Precise) demonstrating significant improvement in midcheek sulcus depth, tear trough reduction, dark eye circles reduction and overall facial contour, indicative of successful volumetric augmentation and tissue remodeling (Images obtained with informed consent from a 45-year-old female patient treated at our center).

When an individual smiles or makes facial expressions, the skin overlying the groove tends to deepen, as the fibrous band of the ligament restricts skin movement, making the groove appear more pronounced.^22^ Clinically, it is often necessary to address not only the groove itself but also the overall volume deficiency in the anterior cheek region during treatment. In some instances, the midcheek groove, which runs diagonally beneath the tear trough deformity, extends upwards to the nasojugal groove, creating a continuous line from the medial to the lateral cheek. This line may appear as a single, uninterrupted groove or as a slightly broken or angled line, depending on the case (Figure 5).^35^, ^36^, ^37^, ^38^, ^39^, ^40^

**Figure 6:**
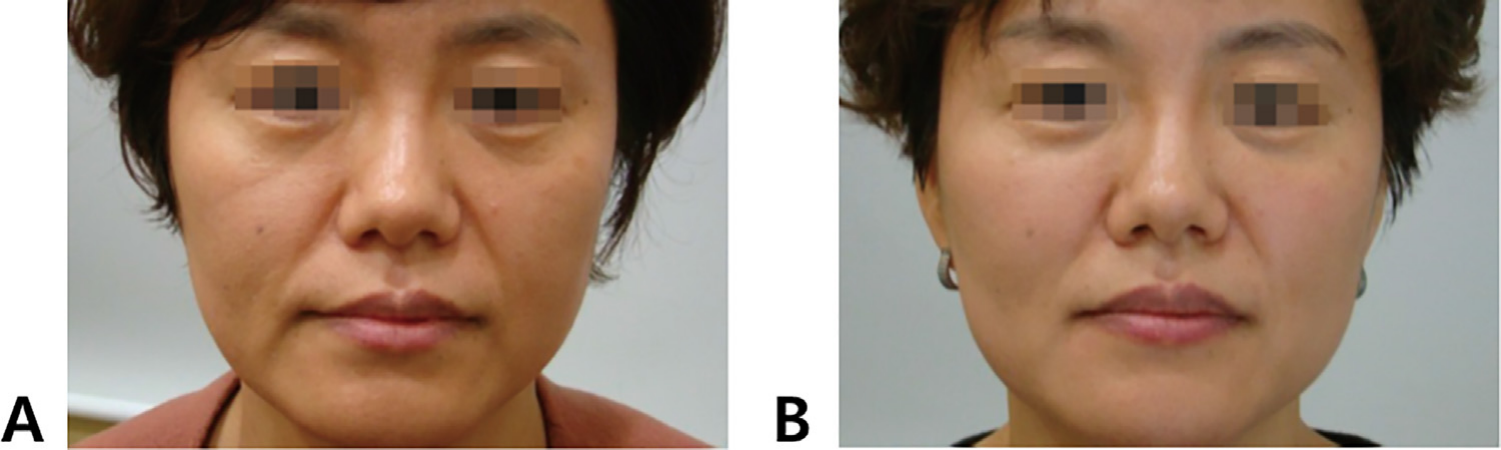
Evaluation of midcheek sulcus and nasojugal groove pre- and post-intervention. Panel A: Pre-intervention state characterized by prominent midcheek sulcus and nasojugal groove. Panel B: Post-intervention outcome illustrating significant amelioration of both aesthetic concerns and an overall enhancement in facial harmony. (Images obtained with informed consent from a 51-year-old female patient treated at our center).

As previously mentioned, in cases where the nasojugal groove is absent or faint, the midcheek groove and tear trough deformity may seem connected, forming a continuous line. However, closer examination often reveals a break or discontinuity in the groove. This break marks the transition from the tear trough ligament in the medial canthal area to the zygomatico-cutaneous ligaments laterally. Therefore, when treating such cases, it is essential to tailor the filler type and injection depth according to the anatomical differences on either side of this break. This approach ensures optimal outcomes by appropriately addressing the unique structural characteristics of both the medial and lateral areas (Figure 6).

#### Procedure method

**Figure 7:**
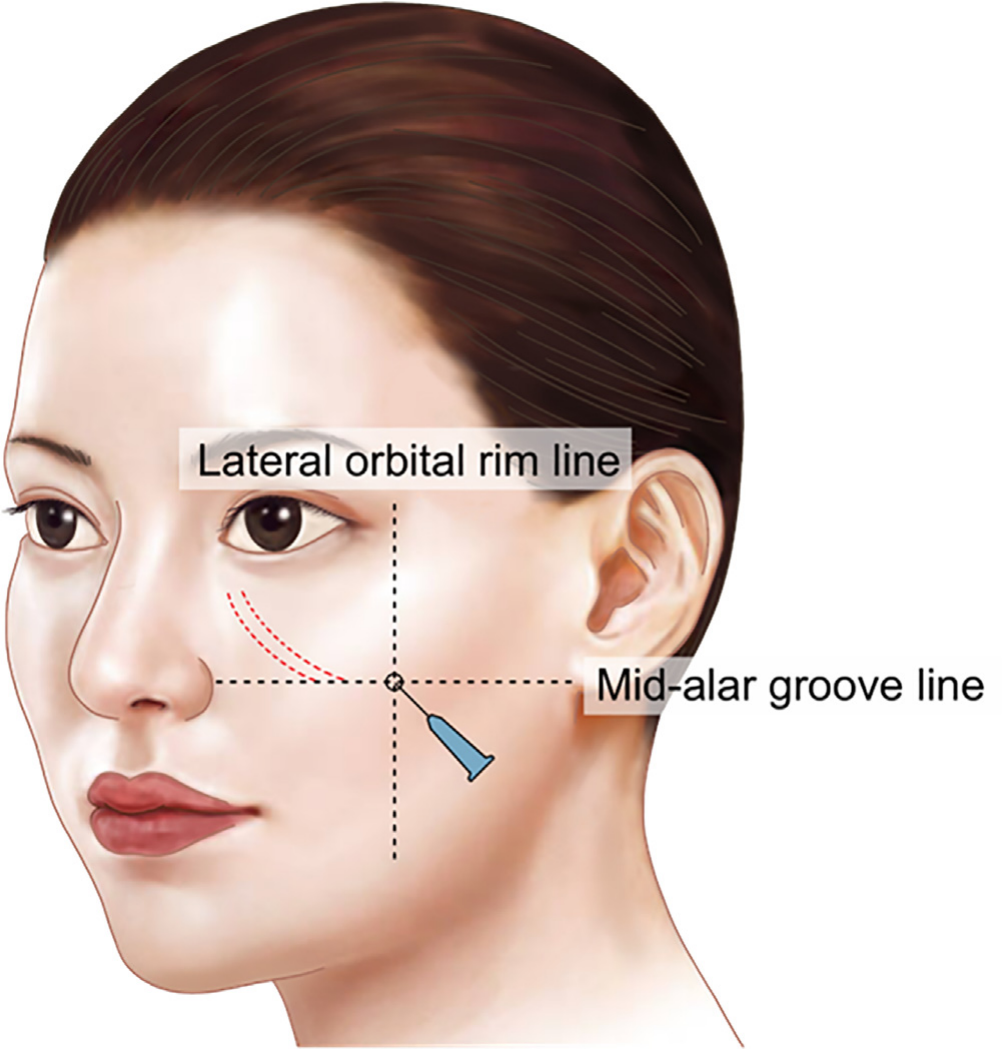
Injection technique and anatomical planes for midfacial rejuvenation. Two entry points are illustrated: *Entry point 1 (medial)* just below the midcheek groove, and *Entry point 2 (lateral)* beneath the malar eminence. Filler is placed in two planes—**deep plane** (SOOF/deep malar fat pad; solid arrow) for structural support, and **superficial plane** (superficial malar fat pad; dashed arrow) for contour refinement. Both follow a **retrograde cannula technique** to achieve balanced midface rejuvenation.

For laterally extended midcheek grooves, a blunt cannula can be used to partially release the dense fibrous bands that contribute to the visible depression, creating space for even filler distribution. Mohammed et al. argued that releasing retaining ligaments during facial aesthetic surgery improves tissue mobilization while using ligaments as anatomical landmarks to avoid nerve injury.^23^ The recommended entry point is just inferior to the midcheek groove at the intersection of a vertical line from the lateral orbital rim and a horizontal line from the midpoint of the alar groove (Figure 7).

Product selection is guided by rheology. . For deep structural support in the SOOF and deep malar fat pad, choose fillers with higher elastic modulus (G′) and higher cohesivity to help resist deformation and maintain projection with the least amount needed. The HA filler used in this paper for this purpose were Maili Volume (21mg/ml, Sinclair). For superficial blending and contour refinement, select fillers with lower G′ and greater elasticity are preferred to conform to mobile, thin tissues while minimizing surface irregularity, particularly in the plane between muscle and dermis. The HA filler used in this paper for this purpose were Maili Precise (15mg/ml, Sinclair) These principles are brand-agnostic and should be matched to the target plane’s biomechanical demand and patient-specific soft-tissue characteristics.

Position the patient in a sitting upright position. When treating the entire midcheek groove, begin by puncturing the entry point with a needle, then insert a 21 G or 23 G cannula. The cannula is tunneled beneath the groove to release the dense fibrous bands, creating sufficient space for even distribution. This technique helps prevent the common issue of the groove remaining depressed while the surrounding areas bulge.

After adequate tunneling and partial release, perform retrograde linear threading and layered augmentation to restore deep support (deep malar/SOOF), with optional superficial refinement in the superficial malar fat pad. Pause after every injection of 0.1-0.2ml to observe the immediate, visible result before continuing, to prevent overfilling. In areas compressed by superficial fibrous bands, small-volume intradermal or subdermal tenting with soft filler can smooth the surface (usage of the thenar eminence with gentle plunger pressure is advised for minute aliquot filling). Final assessment should include dynamic animation (smile) to detect focal pooling along bands and ensure even distribution.

Preventive strategies for overfill and surface irregularity are as important as rescue protocols. These include conservative initial dosing with staged touch-ups, strict adherence to layer-appropriate product selection, injection along rather than perpendicular to high-risk vascular corridors, and systematic photographic documentation to allow objective comparison before and after treatment.^34^, ^40^, ^41^, ^42^

## Discussion

The treatment of dark circles and midcheek grooves represents a significant advancement in non-surgical facial rejuvenation. The paradigm shift toward anatomy-based injection techniques underscores the importance of understanding the layered architecture of the face, particularly the ligamentous and fat compartment structures. This approach enhances predictability, natural outcomes, and safety by guiding filler placement according to biomechanical function and anatomical boundaries rather than arbitrary aesthetic zones.^15^^,^^20^^,^^24^, ^25^, ^26^, ^27^, ^28^, ^29^, ^30^

### Complications and safety considerations

Common adverse effects include transient bruising, edema, nodularity, and the Tyndall effect, especially in thin-skinned patients. Bruising—occurring in approximately 19–24% of treatments—and edema (10–15%) are the most frequent self-limited reactions.^31^ Although rare, severe events such as vascular occlusion (0.001–0.01%) and visual loss (0.0001–0.01%) remain the most serious complications, necessitating rigorous preventive strategies. These include the use of blunt cannulas in high-risk regions, low-pressure injection with small aliquots, aspiration, frequent needle or cannula repositioning, and readiness to administer hyaluronidase in case of vascular compromise or clinically significant overfill. Recognition of angular and infraorbital vascular anastomoses is essential for preventing retinal artery embolization.

### Evidence gaps and methodological limitations

Despite growing adoption of anatomical-based filler techniques, the current evidence base remains limited by small case series, heterogeneity in methodology, and the absence of randomized controlled trials. Most available data are qualitative or expert-opinion level, with minimal quantitative synthesis of complication or success rates. Standardized grading of infraorbital hollows or midcheek grooves, objective volumetric assessment, and long-term follow-up are inconsistently reported. Future studies should incorporate predefined, validated scales (for example, infraorbital hollow severity scales, global aesthetic improvement scales) and objective imaging (three-dimensional surface photography or ultrasound-based thickness measurement) to quantify changes. Prospective registries could also capture complication rates, the need for hyaluronidase or surgical revision, and recurrence patterns across different filler types and injection strategies.^32^

### Comparative and adjunctive modalities

While dermal fillers offer immediate volumization and contour correction, comparative studies suggest that fat grafting provides longer durability at the expense of procedural invasiveness, whereas energy-based devices (laser, RF, ultrasound) yield incremental skin tightening and texture improvement. Integrating HA fillers with fractional laser or radiofrequency microneedling has demonstrated synergistic benefits by simultaneously enhancing dermal quality and restoring volume.^10^, ^11^, ^12^ Surgical options, including blepharoplasty or SOOF repositioning, remain the definitive management for pronounced dermatochalasis or pseudoherniation of orbital fat, underscoring the need for individualized, multimodal treatment planning.

### Ethnic and anatomical variation

Anatomical diversity across populations profoundly influences filler outcomes. Asian patients, in particular, often present with denser retinacular cutis and thicker subcutaneous fibroseptal networks, which limit filler spread and increase resistance to cannula passage.^33^ These structural characteristics necessitate lower injection pressures, smaller aliquots, and more superficial layering to achieve balanced correction without overfilling. Conversely, Caucasian patients generally exhibit looser soft-tissue septa, allowing greater filler diffusion but higher risk of migration. Practitioners should adapt techniques to ethnic morphology rather than applying uniform protocols.

### Emerging technologies

Recent advances such as ultrasound-guided filler delivery and three-dimensional vascular mapping are redefining precision in facial rejuvenation. Ultrasonography allows real-time visualization of vascular structures and filler deposition, improving procedural safety and outcome reproducibility. Moreover, AI-driven imaging and simulation may enable pre-procedure risk assessment and personalized planning of filler rheology and injection vectors, although these tools remain in early clinical adoption.

### Patient-centered outcomes and psychosocial impact

Most published reports on infraorbital and midcheek filler treatment focus on physician-rated scales and photographic examples, while formal patient-reported outcome measures are rarely used. Yet the primary motivation for treatment is subjective improvement in perceived tiredness, attractiveness, and social confidence. Incorporating validated instruments such as FACE-Q or other region-specific questionnaires would allow systematic evaluation of patient satisfaction, quality of life, and psychosocial recovery after treatment or after correction of complications such as overfill. Future prospective studies should integrate these tools alongside objective measures to provide a more holistic assessment of benefit and risk.

## Limitations

This review is narrative in nature and may be subject to selection bias, including restriction to English-language publications and reliance on studies with heterogeneous designs and outcome measures. We did not perform a systematic extraction or pooling of quantitative data, nor did we conduct controlled comparisons between specific injection techniques, filler products, or adjunctive modalities. As a result, the recommendations presented are best interpreted as an evidence-informed synthesis of anatomical and clinical concepts rather than definitive practice guidelines.

Furthermore, most of the anatomical insights are derived from cadaveric or imaging studies, which may not fully capture dynamic soft-tissue behavior or long-term filler migration in vivo. Clinical reports frequently lack standardized grading scales, objective volumetric assessment, or long-term follow-up beyond 6–12 months, limiting the generalisability of outcomes and complication rates. Prospective, controlled clinical studies and registries are needed to validate the anatomical algorithms proposed here and to establish robust, quantitative and patient-centered benchmarks for success.

## Conclusion

Anatomy-based filler techniques for the infraorbital and midcheek regions represent an important evolution in aesthetic medicine when performed with a precise understanding of ligamentous support, fat compartment behavior, and vascular anatomy. By integrating careful pre-treatment diagnosis, layer-appropriate product selection, conservative dosing, and structured safety protocols, practitioners can optimize the balance between rejuvenation and risk.

Standardization of technique, ethnic-specific adaptation, and incorporation of real-time imaging will be critical to improving reproducibility and safety in future applications.

Equally important will be the systematic use of objective imaging, complication registries, and patient-reported outcome measures to move from experience-based practice toward truly evidence-based infraorbital and midcheek rejuvenation.

## Author contributions

All authors have reviewed and approved the article for submission.

## Financial disclosure

There is no financial disclosure to report.

## CRediT authorship contribution statement

**Gi-Woong Hong:** Conceptualization, Writing – original draft, Writing – review & editing, Visualization, Supervision. **Isaac Kai Jie Wong:** Conceptualization. **Jin-Hyun Kim:** Conceptualization. **Kyu-Ho Yi:** Conceptualization, Writing – review & editing, Visualization, Supervision.

## Declaration of competing interest

The authors declare no conflicts of interest.
